# The evolving landscape of restrictive cardiomyopathy treatment: clinical trial trends and future directions

**DOI:** 10.3389/fcvm.2025.1561917

**Published:** 2025-09-24

**Authors:** Bin Deng, Wenhua Liu

**Affiliations:** ^1^ Department of Cardiology, Shenzhen Bao’an Traditional Chinese Medicine Hospital Group, Shenzhen, Guangdong, China; ^2^ Department of Endocrinology, Shenzhen Bao’an Authentic TCM Therapy Hospital, Shenzhen, Guangdong, China

**Keywords:** restrictive cardiomyopathy, transthyretin amyloid cardiomyopathy, clinical trials, therapeutic landscape, gene therapy

## Abstract

Restrictive cardiomyopathy (RCM) is a rare form of heart muscle disease characterized predominantly by diastolic dysfunction and restrictive filling, for which no guideline-supported pharmacological treatment currently exists. We reviewed the clinical trial landscape for RCM to identify emerging therapeutic strategies and trends. Using the TrialTrove database, we identified 63 RCM-related clinical trials (2007–2024) after excluding studies of standard therapies or unrelated conditions. Our analysis shows that research interest in RCM has remained modest but steady, with many trials in early (Phase I) and late (Phase III/IV) stages. Transthyretin stabilizers, particularly tafamidis, accounted for a significant portion of these trials and have demonstrated improved cardiac function and outcomes in transthyretin amyloid cardiomyopathy (ATTR-CM). In addition, novel disease-modifying approaches – including antisense oligonucleotides, RNA interference therapies, and gene-editing strategies – are being explored in clinical trials, reflecting a shift towards targeted treatment of underlying causes. Approximately half of the identified trials have been completed, though a few were terminated early due to insufficient efficacy. These findings highlight a dynamic and evolving therapeutic landscape in RCM. While tafamidis has substantially advanced ATTR-CM management, emerging RNA-silencing and gene therapy techniques hold promise to address the unmet needs in RCM, warranting further large-scale studies to validate their safety and efficacy.

## Introduction

RCM, the least common form of cardiomyopathy, is characterized by severe diastolic dysfunction with restrictive ventricular filling and elevated filling pressures (1). In advanced stages, left ventricular systolic function and chamber size are usually preserved, while marked biatrial enlargement is often observed (2). RCM can be familial or acquired, with secondary causes frequently involving infiltrative or systemic diseases such as cardiac amyloidosis, sarcoidosis, or hemochromatosis (3). At present, no disease-specific pharmacological treatment strategy for RCM is endorsed by clinical guidelines. However, recent advances in biomedical research have led to the development of novel therapeutic approaches, particularly for transthyretin amyloid cardiomyopathy (ATTR-CM) (4), which have gained increasing attention in clinical trials and could challenge conventional supportive treatment regimens.

On October 1, 2024, we conducted a comprehensive search in the Citeline TrialTrove database using the term “Restrictive Cardiomyopathy”. TrialTrove aggregates trial data from major registries (e.g., https://www.ClinicalTrials.gov and WHO ICTRP) with built-in de-duplication and data quality controls; therefore, separate searches of those registries were not performed. Trials were included only if they focused on patients with a confirmed RCM diagnosis and investigational therapies specific to RCM. We excluded trials evaluating only conventional heart failure treatments, those with unspecified trial phase, and any study targeting conditions not strictly classified as RCM (for example, diabetic cardiomyopathy or other diseases exhibiting a restrictive physiology without an RCM diagnosis). Trials at unknown stages or investigating incompatible diseases or therapies were also excluded. A total of 63 clinical trials meeting these criteria were identified and categorized by phase, status, and treatment mechanism to assess trends in RCM drug development (Supplementary Table S1). This study utilized publicly available data and did not require ethical approval or informed consent.

## Results

**Figure 1:**
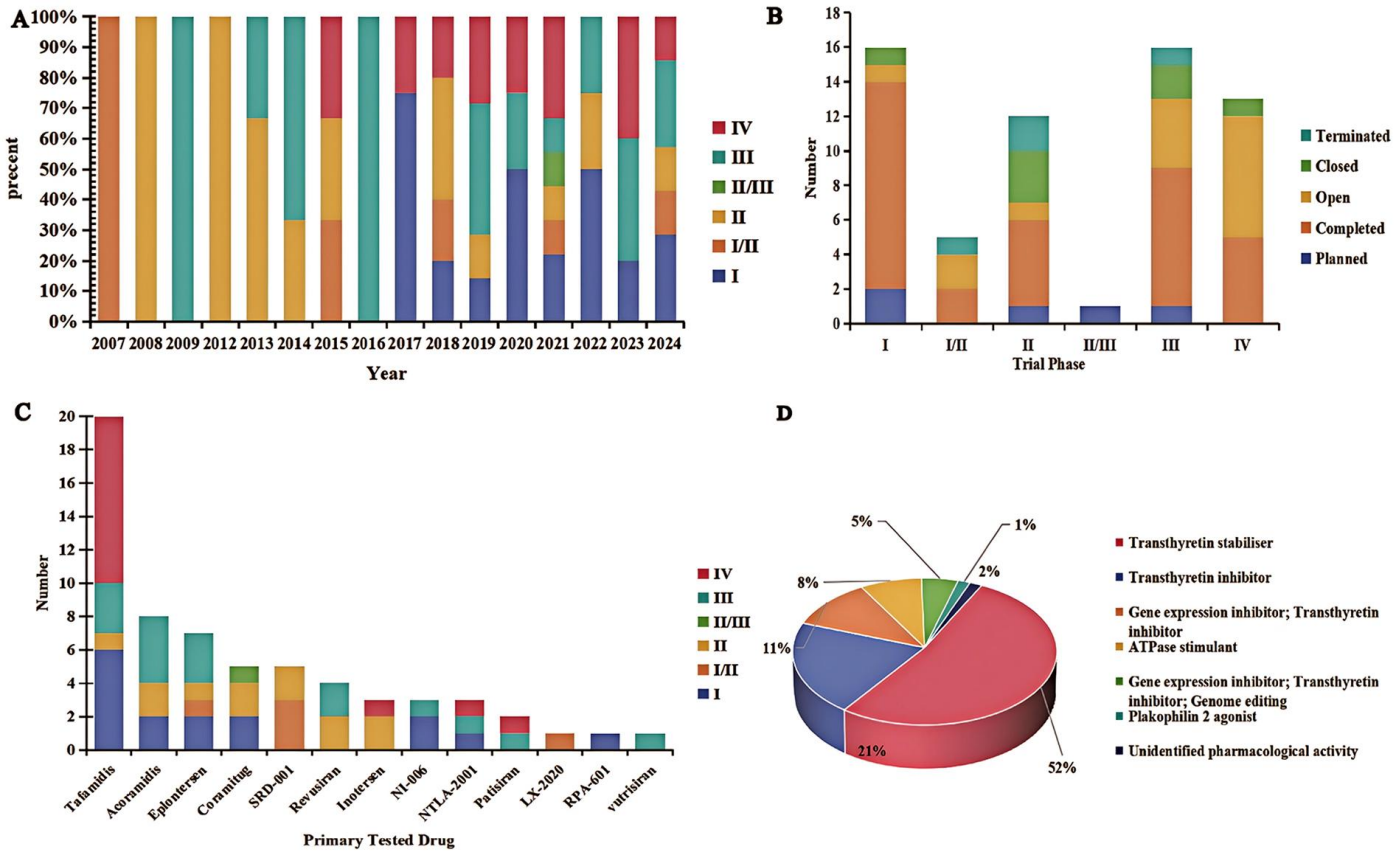
Clinical trial landscape of restrictive cardiomyopathy. **(A)** Geographic distribution and status of RCM clinical trials across different phases, with an increase in trial initiations observed after 2018 as indicated by the trend line. **(B)** Distribution of trial statuses across phases from 2007 to 2024. **(C)** Proportion of trials by therapeutic category in RCM (e.g., TTR stabilizers, gene-silencing agents, etc.). **(D)** Distribution of mechanisms of action of drugs being tested in RCM trials.

From 2007 to 2024, clinical trials investigating pharmacotherapies for RCM have been conducted across eight countries. The annual number of RCM trials has remained relatively stable in recent years, with a slight increase in new trial initiations observed after 2018 (Figure 1A). A substantial proportion of trials are in Phase I and Phase IV, suggesting ongoing active research and a continued need for effective treatments. In terms of trial phase distribution, the majority of trials are in Phase I (23.8%), Phase III (25.4%), and Phase IV (20.6%). Fifteen Phase I trials emphasize the novelty and potential of emerging ATTR-CM therapies, while sixteen Phase III trials aim to validate their efficacy, indicating that the clinical benefits of several investigational drugs are still under evaluation. Thirteen Phase IV trials primarily focus on post-marketing surveillance and long-term safety. Of the 63 trials, 32 (50.8%) have been completed, and 4 (6.3%) were terminated early due to lack of efficacy (Figure 1B).

Tafamidis (5), a transthyretin stabilizer, currently dominates the therapeutic landscape in ATTR-CM, being investigated in approximately one-third of the trials (31.7%). Tafamidis binds to transthyretin and stabilizes the tetramer, preventing its dissociation and subsequent amyloid fibril formation. Published results from Phase I, Phase III, and Phase IV trials have demonstrated tafamidis's ability to significantly reduce myocardial amyloid deposition and improve patients' exercise capacity, cardiac biomarker levels, and overall cardiac function (5). Specifically, patients treated with tafamidis showed improvements in six-minute walk distance, NT-proBNP levels, left ventricular strain, and ejection fraction, corresponding with reductions in mortality and cardiovascular-related hospitalizations in both wild-type and hereditary ATTR-CM populations. While tafamidis is administered orally with a well-established safety profile, newer transthyretin-targeted therapies offer alternative mechanisms and routes of delivery. For example, the Phase III APOLLO-B study of patisiran (an siRNA therapeutic) demonstrated that patisiran maintained six-minute walk distance and preserved quality of life in ATTR-CM patients compared to placebo (6), albeit with periodic infusions and a risk of infusion-related reactions (6). Likewise, inotersen has shown the ability to improve cardiac function and reduce cardiac amyloid burden in patients, though as an antisense oligonucleotide it necessitates regular subcutaneous injections and careful monitoring for adverse effects (7). By contrast, tafamidis's convenient oral dosing and long-term tolerability remain advantages in clinical practice. Another TTR stabilizer, acoramidis, achieved significant increases in transthyretin levels along with reductions in mortality and heart-failure hospitalizations in its Phase III trial, indicating a magnitude of benefit comparable to tafamidis.

Other therapeutic approaches are also under active investigation. Inotersen, NTLA-2001, and patisiran are examples of agents currently in Phase III or Phase IV trials. Inotersen is an antisense oligonucleotide that binds to TTR mRNA via an RNaseH1-dependent mechanism, leading to degradation of mutant and wild-type TTR mRNA and thereby preventing transthyretin protein production. Phase IV results suggest that inotersen can improve cardiac function, reduce cardiac amyloid burden, and potentially enhance quality of life and survival in ATTR-CM patients (7). However, as an ASO therapy, inotersen requires ongoing injections and safety monitoring, in contrast to the oral administration of tafamidis. Additional investigational agents – including acoramidis, eplontersen, coramitug, revusiran, vutrisiran, and NI-006 – are primarily in Phase II or Phase III trials. For instance, the Phase III trial of acoramidis (AG10, another TTR stabilizer) showed that treatment led to significant increases in serum TTR levels by Day 28, which were associated with reduced all-cause and cardiovascular mortality and fewer heart failure hospitalizations (8). Moreover, acoramidis therapy significantly improved a composite endpoint of cardiovascular death and hospitalization, with clear benefits evident as early as three months post-treatment initiation (9). These outcomes suggest that acoramidis can deliver clinical efficacy on par with tafamidis, with a similarly favorable safety profile observed in trials. Gene therapy-based interventions are also being explored: agents delivered via adeno-associated viruses (AAVrh10) such as LX-2020 and RP-A601, as well as a gene therapy targeting sarcoplasmic/endoplasmic reticulum Ca^2+^-ATPase (SRD-001), have reached Phase I or II trials. Notably, SRD-001 was discontinued in early development due to insufficient efficacy (Figures 1C,D).

## Discussion

Our analysis underscores that ATTR cardiomyopathy dominates the RCM trial landscape. Over 90% of registered trials focus on transthyretin-related (ATTR) amyloidosis of the heart, reflecting its high prevalence and the advent of effective therapies (e.g., tafamidis, patisiran) (Maurer et al.) (10). This emphasis means other RCM causes receive little attention: for example, cardiac sarcoidosis (CS) has no prospective clinical trials to guide management, and similar gaps exist for iron-overload or idiopathic RCM. In practice, these diseases rely on established treatments (e.g., immunosuppression in CS, chelation for iron) and small case series, making large randomized trials impractical or unprofitable. The imbalance implies that progress in RCM is largely driven by ATTR, while non-ATTR subtypes remain under-studied.

Among ATTR-directed therapies, two small-molecule stabilizers and three genetic silencers define the current era. Tafamidis is an oral TTR stabilizer that in the ATTR-ACT trial significantly reduced mortality and hospitalizations in ATTR-CM (hazard ratio for all-cause death ∼0.70) (Maurer et al.) (10). It is generally well tolerated with a favorable safety profile (10). Acoramidis (oral AG10) is a next-generation stabilizer that in ATTRibute-CM produced a significant improvement in a hierarchical endpoint of death, hospitalizations, biomarkers, and functional capacity (win ratio ∼1.8) (10); it likewise showed no new safety signals (11). Patisiran is an intravenous RNAi agent (siRNA) dosed every 3 weeks; in the APOLLO-B trial it preserved 6-minute-walk distance and quality-of-life compared to placebo over 12 months, with an encouraging safety/tolerability profile (12). Vutrisiran is a subcutaneously administered siRNA (once every 3 months) that rapidly silences TTR; in HELIOS-B it dramatically reduced the risk of death or cardiovascular events (≈37% lower risk than placebo) and sustained quality-of-life gains over 30–36 months, with a long-term safety profile similar to patisiran (13). In contrast, inotersen is a weekly subcutaneous antisense oligonucleotide approved for hereditary ATTR with neuropathy. In ATTR neuropathy trials it improved neurologic progression and quality-of-life (Benson et al.) (9), but it requires intensive monitoring for thrombocytopenia and glomerulonephritis as notable toxicities (14).

Continued efforts through large-scale, long-term clinical trials are essential to validate both existing and emerging therapies for RCM. Improvements in early diagnostic technologies may also enhance patient prognosis by enabling timely and appropriate intervention. Looking to the future, the therapeutic landscape for RCM is expected to become more personalized and effective with the continued development of precision medicine, gene therapies, and cell-based therapies, which could significantly improve outcomes for patients with this challenging condition.

## Data Availability

The original contributions presented in the study are included in the article/Supplementary Material, further inquiries can be directed to the corresponding author.
